# AI to review government records: new work to unlock historically significant digital records

**DOI:** 10.1007/s00146-025-02221-0

**Published:** 2025-02-22

**Authors:** David Canning, Lise Jaillant

**Affiliations:** 1 Cabinet Office, London and Manchester, UK; 2 Loughborough University, Loughborough, UK

**Keywords:** Born-digital records, Digitised records, Artificial Intelligence, Appraisal, Records lifecycle, Records disposal

Government professionals need to work closely with archival institutions to make sure that important born-digital records are identified and preserved in the short term, before being transferred for long-term archiving and access. Knowledge and information (KIM) professionals in government and archivists face similar challenges. In the UK, government records (in digital and paper form) must by law be transferred to archival institutions after 20 years. In the USA, the National Archives and Records Administration issued a memorandum in December 2022, stating that “after June 30, 2024, NARA will no longer accept transfers of permanent or temporary records in analog formats and will accept records only in an electronic format with appropriate metadata.”^1^

These masses of born-digital government records must be reviewed to select historically significant documents for preservation, and delete ephemeral information.^2^ This process of appraisal cannot be done manually. Given the volume and complexity of digital information, and the rate at which digital information is being created, using computational appraisals such as Artificial Intelligence is a no longer a choice, but a necessity. Without automation, the risk is that the number of unstructured and unmanaged records keeps growing (these records, and their exponential rise, are often referred to as “digital heaps”). The lack of discoverability and accessibility can lead to a lack of transparency and accountability, and to legal liability in case of freedom of information (FOI) requests^3^ or public inquiries. In the long term, the inability to find, select and transfer important records has an impact on the historical archive available to researchers and other users in archival institutions.

This article presents groundbreaking new work on AI applied to the government records of the Cabinet Office, to identify historically significant records to preserve and other records that can be deleted. The Cabinet Office is the UK central government department that supports the Prime Minister in the effective running of government. In partnership with the HM Treasury, it is the corporate headquarters for government, and takes the lead on certain critical policy areas. Its records are among the most important that are deposited in The National Archives, covering those of the Prime Minister, Cabinet proceedings, government efficiency and reform and the formulation of legislation, among other areas.

The significance of this article goes well beyond UK central government. Indeed, this successful experimental work can be deployed in other contexts to select and unlock digital records with historical value. The innovative methodological approach we describe provides a step change in progress in the archives and records management sector. This article does not only describe a radically new methodology to appraise digital records, but also makes a significant theoretical contribution to academic disciplines that seldom talk to each other—including archival studies, digital humanities and computer/ data science. Thirty years ago, Terry Cook wrote: “Archival mindsets and solutions reflect generations of sound practice in a paper-based world.” He asked: “How do we recast our paper minds to deal electronic realities?” (Cook 1994). This article discusses how moving from paper to digital records management has presented considerable challenges to traditional archives and records management practice, and created barriers to accessing records in archives. It proposes a new archival mindset that reflects the digital realities faced by records managers, archivists and users. This theoretical approach combines distant viewing (necessary to approach huge amounts of records) and close reading of a selection of records (to ensure the effectiveness of computational approaches). This combination of “distant” and “close” is an answer to the need to move away from paper minds and embrace digital archival realities and methodologies.^4^

The application of AI to digital records at the Cabinet Office (CO) required a rigorous and ethical process, which was achieved thanks to cross-disciplinary and cross-sector collaborations. David Canning, Head of Digital KIM at the CO, led a team with a wide range of expert competencies—such as the ability to develop algorithmic and mathematical models. He also worked with colleagues in other branches of government—including with the research team at The National Archives,^5^ who reviewed the algorithms used for the selection of archival records. This article was co-written with Lise Jaillant, an academic with expertise in digital humanities and extensive experience of international projects on AI applied to archives. Jaillant’s viewpoint as a user of archives brought a much-needed perspective. Indeed, the scholarship and practical experiments on AI and archives are often led by archivists and records managers, with little input from users. This article shows the need for engagement across the archival circle—from record creators to archivists, and from archivists to users.

The article is divided into three parts. The first section explores the reasons that gave rise to the accumulation of “digital heaps” in government departments that have the effect of inhibiting transparency and preventing good archival organisation. The second section discusses the importance of implementing a digital mindset to solve the problem of unstructured digital records. This implies considering human behaviour when designing a system to manage the digital information life cycle.

In addition to human behaviours, records management systems need to include good information governance, risk management and the use of institutional levers to promote best practice. To solve the challenge of “digital heaps,” computational approaches are also essential. The third section starts with an overview of current work on AI applied to archives. It then explains the digital disposal methodology implemented at the Cabinet Office. It also explains the measures taken to change the cultural and behavioural aspects that prevent good record keeping and can lead to important records not reaching archives. Finally, the conclusion explores where records management might go next in deploying Artificial Intelligence technologies and the benefits these may have in opening up archives for future historians. Recommendations are offered to facilitate the ethical application of AI to records and archives, in the government sector and beyond.

## Understanding the rise of the “digital heap”

### The records life cycle and legal obligations

In his treatise on the management of modern archives, T. R. Schellenberg (1956) referred to the concept of the records life cycle, in which records (which includes information) are created, recorded, managed and then either destroyed or permanently retained through a process of disposition. The legislative framework within which UK public bodies operate reflects this concept: The Public Records Act 1958^6^ [Sect. 3(1)] requires government departments to select records for permanent preservation, ensure their safe keeping and then arrange for their transfer to The National Archives 20 years after their creation [Sect. 3 (4)]. The Data Protection Act 2018, which placed the European General Data Protection Regulation onto the UK statute, requires organisations that process personal data to only retain data for as long as it can reasonably assert a legal basis for doing so. Data controllers are therefore required to apply rules for the length of time data should be retained, as well as a system of review. The Secretary of State’s Code of Practice on the Management of Records, issued under Sect. 46 of the Freedom of Information Act 2000,^7^requires the public authorities to which it applies to:

2.1.3. … periodically assess the information they hold. They must know why they are keeping it and must also be able to explain why they no longer hold information, by keeping a record of decisions to keep, archive or destroy information.

While the Public Records Act has been in force for seven decades, the provisions of the Data Protection Act and Code of Practice are relatively new updates to preceding law, which required modernising to keep up with developments in technology. The rationale behind the introduction of the European General Data Protection Regulation in 2018 was to update a Directive made 20 years earlier due to the rapid development of cloud computing, social media and Internet-based transactional services.^8^ A first version of the Code of Practice was issued in 2001 when most public authorities were still employing paper-based methods of record keeping. The updated version, issued in 2021, included a new requirement for public authorities to consult the person responsible for records management in the organisation, before designing, developing or procuring IT systems and applications. This provision was a direct response to records managers’ complaints that technology, and the way it had been deployed, was impeding good practice, and, at worst, was preventing compliance with legal obligations.

### Systemic failure

Since Microsoft Office products began to be widely adopted in the 1990s, the rapid pace of technology advancement and the impact this has had on digital record keeping have been profound.

Microsoft Office applications were initially used by many to replace the dictation and typing up of hard copy documents, and documents created digitally were printed off and stored in physical files. As the idea of the digital office took off in the mid-2000s, electronic document and records management systems (EDRMS) were introduced as an attempt to replace physical filing. It was believed, perhaps reasonably at the time, that people would “declare” the final version of a document by moving a file from its place of creation to a records repository, and delete earlier versions and drafts. This is not what happened in practice: numerous versions of documents often remained on servers. Poor recordkeeping practices have led to significant challenges for the creation, management and accessibility of government archives.

In 2015, the UK government commissioned the senior civil servant Sir Alex Allan to carry out a review of government digital records and archives. In his report, Sir Alex wrote:

Existing systems which require individual users to identify documents that should constitute official records, and then to save them into an EDRMS *[electronic document and records management system]* or corporate file plan, have not worked well. The processes have been burdensome and compliance poor. As a result, almost all departments have a mass of digital data stored on shared drives that is poorly organised and indexed.

Furthermore, he also concluded that:

The principles for selection and retention of digital records . . . are well understood. But significant practical problems remain, not least because of the poor organisation of much digital data.

The Government’s official response to this review, published in 2017, accepted all of its conclusions and recommendations, and acknowledged that there had been a collective and systemic failure. Importantly, it laid down a challenge for departments to find solutions to the issues Sir Alex had identified. This included exploring search and analytics technologies since they “can help make sense of large and unstructured information collections” (Cabinet Office 2017).

##  Moving to a digital mindset

What are the reasons for the acknowledged failures in the UK government’s records management? These failures are not unique, and not simply due to poor organisation or management. A new system of working had been created along with supporting technologies to accommodate the so-called “paperless office,” but these largely failed to take into account or correctly predict human behaviour and the way in which the nature of digital systems was unknown. Faced with burdensome processes, busy people will, unless forced, avoid doing tasks that appear optional, and where the benefits of the action are not immediately felt, even if they know that doing so places them in a position of non-compliance.

Indeed, if doing the correct and proper thing would either cause a person inconvenience or place them at a competitive disadvantage to others, they will likely not do it. Consequently, a lack of levers to force appropriate behaviours will frequently lead to non-compliance. This form of systemic failure has a direct impact on records, resulting in an impoverished historic archive. Since human actions can undermine an organisational system through selfish actions, we need to make sure that humans are incentivised appropriately to operate in the best interests of the entire system. In other words, the way in which humans are required to interact with digital systems needs to make compliance easy. This would ideally include removing laborious and repetitive tasks from busy people, changing established business processes where necessary to allow technology to pick up the burden.

### The digital paradigm

In his report, Sir Alex Allan coined the phrase, the “digital heap,” to describe the exponential rise in digital records resulting from poor or non-existent life cycle management (Allan 2015). This includes a pile of legacy digital documents, as well as emails, which range from ephemeral conversations to documents of great historic importance. This term has been adopted across the knowledge and information management sector (Bath 2023), adding to a large corpus of work on appraisal in the digital age (Moss and Endicott-Popovsky 2015; Yeo 2018).

In the context of the Cabinet Office records, there are four main features of the digital paradigm that present a management challenge:

**Volume:** It will vary, but at the Cabinet Office, the volume of digital records is around 1000 times higher than for paper. People have always created several versions of the same document until settling on a final one, but instead of going into the waste paper basket, in digital systems everything created would be retained, including various abandoned drafts. But the biggest contributor to overall volume is currently email, which makes up around three-quarters of the total data volume. The rule of thumb for working out how many pages of text there are for a given data size is circa. 65,000 pages per gigabyte for a document and 100,000 pages for email.^9^ The CO holds around 199 terabytes of documents and emails, and even at a conservative estimate, this translates to at least 12.9 billion pages of text, a pile that grows by about 8% per annum.

File plan: in the world of paper records management, documents are created and placed in files, usually in some sort of chronological order. Papers do get misfiled, but broadly one would expect to find the relevant documents on a given subject contained within a single file. In the digital world, documents relating to a subject may be distributed across a file plan and mixed up with several other subjects. The file plan may therefore result in documents and information not appearing in a logical or chronological order.

Format: digital records can be in hundreds of different formats, such as Office documents, emails, images, PDF or messages. These records can be distributed across not just a file plan, but across several repositories.

Corruption: the Digital Preservation Coalition^10^ points to the fragility of digital data. Metadata is essential for understanding the age and content of digital collections, but it is easily corrupted through the careless replatforming of systems, data transfer or corruption, and is often therefore absent or unreliable. Digital data is also not immutable forever as bit decay, format corruption and obsolescence can all place digital records at a risk of loss.

Records managers face challenging questions about the location and nature of digital records. They need to understand what they hold, the value of that to the official record and its potential historic relevance to archives. They must also record and account for any decisions made relating to disposal. Indeed, decisions to destroy records must be recorded and defensible in case they are called to account by inquiries or courts of law. Transparency is vital to ensure that the government retains public trust in its decision-making and can explain why some records are retained whilst others are destroyed.

Those who create and manage IT systems often appear to believe in destroying almost nothing. However, disposal is essential to responsibly manage records. In a digital corpus so large and complex, life cycle management cannot easily be achieved without the injection of huge resources over such a long period that makes human review impractical. Disposal of digital information must therefore be a clear target for the application of Artificial Intelligence.

The problem to be solved is how to replicate a human decision-making process that would be at least as good as traditional records review methods. For reviewers of digital records at the Cabinet Office, the issue is that the context of digital records is radically different from paper records. The usual points of reference that would help a reviewer make a decision about the value of a record are less obvious or accessible because of the digital paradigm explained above. Consequently, the value of a digital record must sometimes be assessed in isolation if the context of file plan and metadata is poor. Since records are now “born digital,” approaches to their management and preservation need to be digitally fit for purpose. The systemic failure that had contributed to the digital heap happened because of the attempt to solve a digital problem through applying an analogue solution, reproducing a paper-based process in digital form. The move towards computational approaches therefore requires much more than new tools: it requires a digital mindset able to navigate various scales (from “distant” approaches necessary to make sense of huge numbers of records, to “close” reading of records that require human review).

##  Applying Artificial Intelligence to records and archives

The Cabinet Office’s experiment with AI applied to government records is situated within a larger context of increased interest in computational approaches to select, search and use digital archives. This section starts with a short history of AI and archives and the scholarship related to this field. The overall objective is to show that the CO’s project builds on previous work, which has influenced the research methodology, data gathering and assumptions around which the study is based.

Although Artificial Intelligence is often presented as a new technology, research on AI has been going on since the mid-1950s. Dartmouth College in the USA hosted the seminal Dartmouth Summer Research Project on Artificial Intelligence in 1956. This workshop is widely considered to be the founding event of the research field of AI (Cordeschi 2007). In the library sector, there was a period of experimentation with AI from the mid-1980s through the mid-1990s that mainly focused on the use of expert systems to assist human decisions (Hewitt 1988; Aluri and Riggs 1990). The current interest on AI applied to archives dates from the 2010s. The first projects focused on a specific AI technology—such as HTR (Handwritten Text Recognition) in the case of Transkribus, a platform for the text and image analysis of historical documents. The platform was created in the context of the two EU-funded projects (2013–2019), and it has since been developed by the cooperative READ-COOP, but Transkribus remained an outlier, at a time when Artificial Intelligence was not a term well known in the archive and records management sectors.

It was only in the late 2010s and early 2020s that the field of archives and AI emerged, with the creation of transnational and cross-disciplinary networks, developed in response to the lack of forums for this kind of innovative research. These networks did not limit themselves to a specific AI tool or function. Their ambition was to develop institutions that would be welcoming to the new field and ensure its sustainability. Lise Jaillant was the first in the UK/ Europe to develop international networks to connect humanities scholars, archivists and computer scientists on both sides of the Atlantic. This work has led to a series of journal special issues,^11^ articles and edited collections (Jaillant 2022a, 2022b; Jaillant and Aske 2024; Jaillant and Caputo 2022; Jaillant and Rees 2023; Jaillant et al. 2025). The perspectives of humanities and other users, and the issue of inaccessible archives, have been central to these networks.

Other important projects have offered complementary perspectives. Focusing on the GLAM sector, AI4LAM curates a list of resources, projects, and tools for using AI in libraries, archives and museums.^12^ In addition, the new discipline of computational archival science (CAS) originated in North America to bring together perspectives from computer science and archival science (through the work of Richard Marciano and Victoria Lemieux in particular). CAS has been defined as:

A transdisciplinary field concerned with the application of computational methods and resources to large-scale records/archives processing, analysis, storage, long-term preservation, and access, with the aim of improving efficiency, productivity and precision in support of appraisal, arrangement and description, preservation and access decisions, and engaging and undertaking research with archival materials (Marciano et al. 2019).

Victoria Lemieux, a professor of archival science based at the University of British Columbia in Canada, has been central to the CAS group, and is also very active in InterPARES Trust AI (2021–2026), “a multi-national interdisciplinary project aiming to design, develop, and leverage Artificial Intelligence to support the ongoing availability and accessibility of trustworthy public records.”^13^ While “access” and “accessibility” are mentioned in the definitions of CAS and InterPARES Trust AI, the main focus has been on records managers and archivists (who make decisions on accessibility) rather than on end users (who need access to produce new knowledge).

Applying AI to records and archives remains largely at the stage of experiments or even vague plans based on the perceived value of automation. Modiba (2022) researched the question amongst the archivist community, and a majority of these archivists believed that AI would be able to assist with disposal. Indeed, respondents were convinced that AI would be able to operate faster than human operators and be able to accurately classify records. Modiba concluded in his framework for the usability of AI in improving records management activities that, “records of value will robotically be moved to the digital archives for archival purpose.” Franks (2022) also proposed that the “automatic classification of electronic records is necessary to address the brewing crisis in the recordkeeping discipline, caused by escalating data volumes and digital rights legislation.” He argued that the classification of records by metadata was becoming unfeasible due to their increasing complexity combined with a growing lack of metadata, and that text classification showed promise as an alternative.

Text classification is a machine learning technique that uses AI to categorise text into predefined groups. Recent experiments with this technique have shown its potential to unlock government records and archives. Graham McDonald (2021) has argued that a text classification approach can be deployed to assist the sensitivity review of born-digital government documents. In particular, this approach can serve to “identify the vocabulary, syntactic and semantic document features that are reliable indicators of sensitive or non-sensitive text.” Once identified, non-sensitive materials can be made available to the public, while sensitive records remain exempt from disclosure.

Likewise, Jason Baron et al. (2022) have studied the case of archival materials exempt from disclosure under the Freedom of Information Act (FOIA), due to issues such as national security or privacy. In the USA as in many other countries, the process of reviewing these documents is manual, leading to backlogs and long delays to public access to the records of their government. Their article shows that the text classification techniques can automatically identify those materials that are exempt from release. Using supervised machine learning, these classifiers can flag sensitive materials, but do not entirely replace human reviewers. Rather, they “would assist reviewers in determining which records to review on the ‘front end’ of any overall review effort.”

Other articles show the usefulness of text classification to identify sensitive materials (and make non-sensitive materials more accessible). In their article on sensitivity classification, Mahmoud F. Sayed et al (2022) note that it is frequent for useful digital content to become inaccessible simply because it is intermixed with sensitive content. Their research relies on classifiers using only words and word sequences. In the future, they add that “additional features such as relationship graphs and temporal patterns might help to further improve classification accuracy.”

The Cabinet Office began experimenting with text classification in 2018 using a data classification tool, called DataLift, which was developed by Automated Intelligence Ltd initially as a tool for cleansing data when migrating it from on-premise to cloud storage. This was used for its ability to index metadata and execute policy rules based on analysis (for example, it could be used to identify and delete digital files of a specified format). This early experimentation showed promise, but it was not until 2021 that funding was received to develop theory into practical application. In 2022, an experimental version of an AI solution that uses text and format classification was successfully deployed to sift and sort records; the system went into production in April 2023 and is now a key component of the CO’s system for managing the life cycle of digital records and populating its digital archive. This production solution relied on an updated version of the DataLift tool, combined with Elasticsearch,^14^ which was used to run the text queries.

When reviewers read Cabinet Office records to judge their potential historic value they are looking at:

- What is the information about, is it for example international diplomacy or some internal administrative issue?
- Who is involved, who is mentioned in the text, who created it and who was it received by?
- Format, as this provides clues as to why the record was created and its function.

From this, we were able to hypothesise that the majority of records of value in the Cabinet Office were most likely to exist in certain formats, and ephemeral, redundant, outdated and trivial (“ROT”) information also existed in certain formats. This led to the creation of a set of rules:

- Word processor formats are assumed to contain information of value, unless the content of the text demonstrates the opposite.
- PDF and email formats (when saved into a document repository) are assumed to be of value because a person has taken the trouble to preserve them.
- Spreadsheets and presentation slides are assumed not to be of value unless the content says otherwise.
- Image and video files are not valuable unless specifically labelled for permanent preservation.

**Fig. 1:**
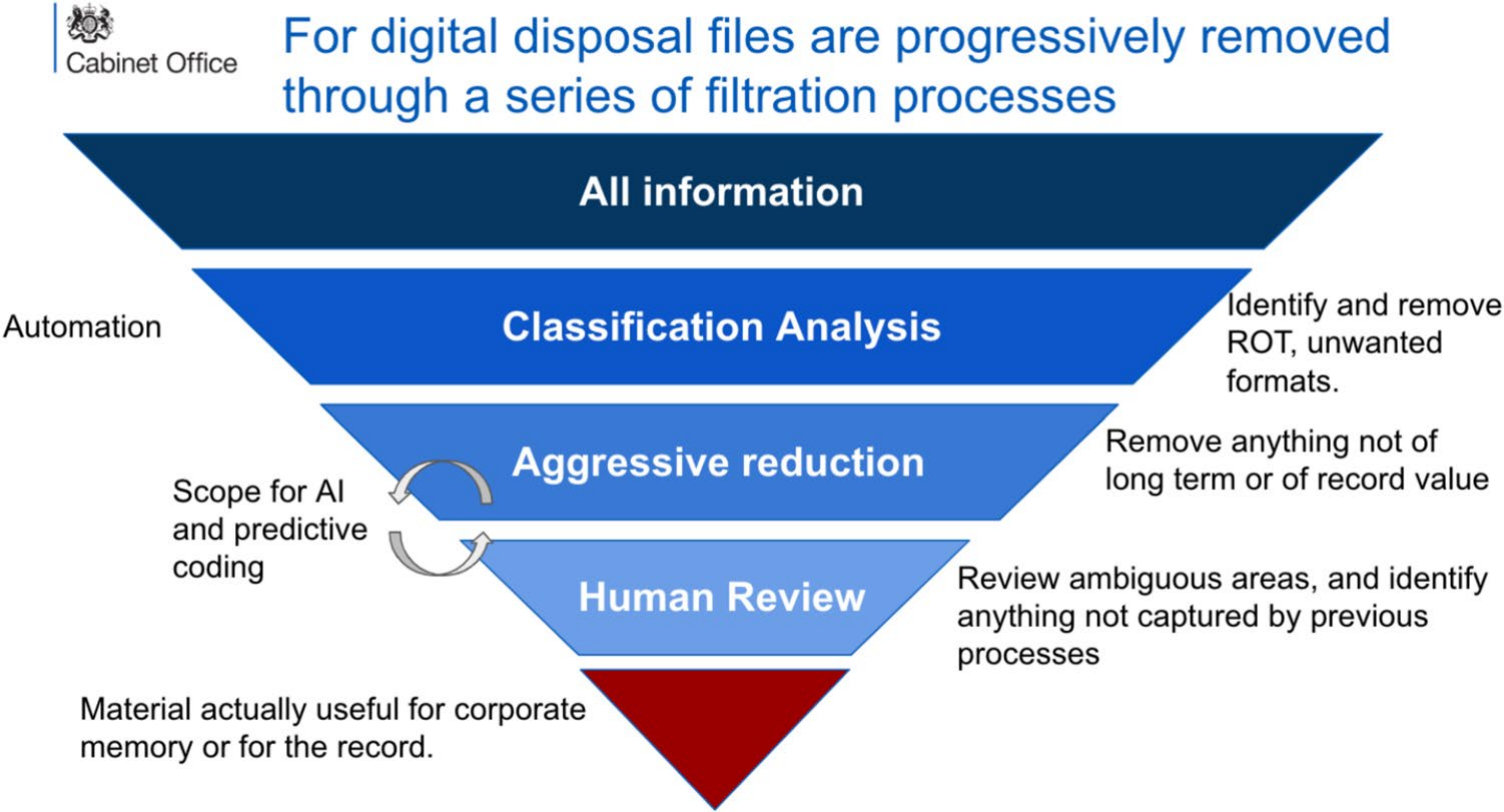
Digital records disposal methodology

The importance of this approach is that it does not rely on record creators adding metadata tags, metadata being present (although it is always very useful) or people declaring records. As long as people can be trained to create records in the right place, and the location of all the records is known,^15^ the system can be run over any collection (Fig. 1).

These rules were developed by analysis of the content of legacy records combined with tacit knowledge and experience. Other organisations will differ in their approach, but in Cabinet Office, for example, PDF files are generally how signed ministerial correspondence is preserved and image files are generally only used to illustrate presentation slides, the exception being photographs from the Prime Minister’s official photographer which are specifically preserved.

Starting in 2018, the CO developed an understanding of the keywords and phrases that are commonly present in its collections. These were derived largely from tacit knowledge of the terms used in the civil service, words and phrases discovered in documents that appeared to denote value or the opposite. A standard taxonomy was also used in some cases. Examples of terms that we recognise as usually indicating value include: “submission,” “minister,” “inquiry,” “bilateral,” “agencies,” “foreign policy” and proper nouns, such as “Richard Wilson” a former Cabinet Secretary, or the names of international leaders. Names require some thought to be useful, however. Should the name “Putin” appear in our records, then it will be obvious who this is about, but the name “Brown,” being a common English name results in too many false positives, so this had to be replaced with “Gordon Brown” the full name of a former UK Prime Minister. We were also able to identify language patterns that indicate ROT, such as “annual leave,” “blah blah,” “Christmas card” and “stuff.” Trial and error involved inputting keywords into the search engine and assessing what came up in the results. Through this process of sampling and testing, it was identified that there are certain circumstances where unimportant files such as templates were being repeatedly flagged as valuable due to them also including value terms, and also out of office messages, or staff timesheets due to the presence of an excessive volume of many proper nouns. The list of terms and phrases is a language model that we collectively refer to as the “Lexicon.”

To mitigate this risk of misclassification, a third category was added which weights terms more strongly, so in files where value terms are combined with ROT terms, the model is more likely to correctly determine value. The hypothesis that some file formats are more likely to contain information of value than others was validated through experimentation, and extension type could then be added as a criteria when weighting the algorithm that drives the AI. A bias towards retention of files with extension types known to be more likely to contain information of value, and conversely, a bias towards deletion of files with formats that our testing showed contain a maximum of < 1% files of value were added. Adding these biases increased the accuracy of our model.

**Fig. 2:**
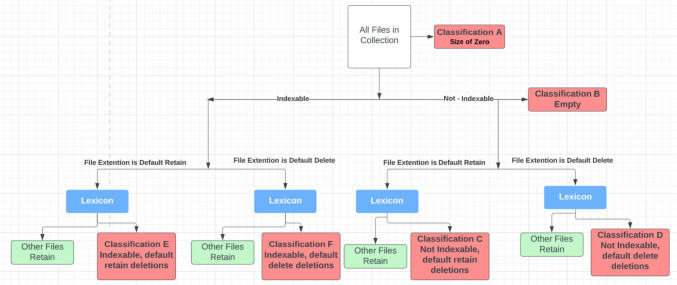
The algorithmic model

Figure 2 is a visual representation of how the algorithm operates. First, empty and size zero files are removed. The remaining files are then divided into indexable file formats (e.g. msg,.doc, etc.) and non-indexable files (images, videos, certain legacy databases, etc.). The next step is to divide the files by file types, dependent on whether we wish to apply a bias towards retention or deletion. The Lexicon of weighted terms are then applied to the four different file “buckets” and further divided into the eight classifications: four of which are for retention and four for deletion. Human reviewers can then check the outcomes and through a process of dip sampling satisfy themselves that the algorithm and Lexicon are operating as expected. Once these checks are completed, the files flagged for retention are moved into the archive.

The Lexicon utilises Elasticsearch to scan the content of files under review and three further methods outlined below were also employed for weighting the relevance of the terms in the Lexicon, adding richer context to the model’s decision-making process in future and supporting governance over how it is used. This includes:

1. *Term frequency (TF)*: the more times that a search term (a word or phrase) appears in the field (file path, other metadata or the content) being searched, the more relevant the document is.
2. *Inverse document frequency (IDF)*: the more documents contain a search term in the field, the less important that term is.
3. *Field length*: if a document contains a search term in a field that is very short (i.e. has few words), it is more likely to be relevant than a document that contains a search term in a field that is very long (i.e. has many words).

### Testing the algorithm

The approach could be described as an intelligence led, risk-based approach. Baselines were established through piloting to understand how the system performs in comparison to non-automated (i.e. entirely human) processes, and what level of reliability can normally be expected. In the pilot, the method used to test how effective the Lexicon was to label a sample of files selected through systematic random sampling. This was considered to be the most efficient method of achieving a statistically significant sample, as the total volume of the files in the collection used for the pilot was in excess of 11 million. One thousand files were selected from the entire corpus of 11.8 million files at equal intervals of 11,800 to ensure that the sample was representative. The percentage of files incorrectly identified through this process provided an estimate for the potential error rate of the system.

The algorithm may make two types of error: incorrectly recommending the destruction of a file that should be retained (Type 1 error, a false positive) and incorrectly recommending the retention of a file that we could dispose of (Type 2 error, a false negative).

**Table:** 

A file is …	Historically valuable	R.O.T
Recommended for deletion	Type 1 error (false positive)	Correctly Identified R.O.T file
Recommended for retention	Correctly identified valuable file	Type 2 error (false negative)

$$\text{Type }1\text{ error }(\text{false positive}) = \frac{number of historically valuable files recommended for deletion}{ total number of historically valuable files in sample}\times 100. \%$$

$$\text{Type }2\text{ error }\left(\text{false negative}\right)= \frac{number of R.O.T files recommended for retention}{ total number of R.O.T files in sample}\times 100. \%$$

### Risk mitigations

In introducing a new, digitally focused, methodology, the risk dynamics has to be changed. Consequently, there was a need to understand and assess this risk and develop appropriate mitigations. The risks were identified as:

- *Transparency*: AI and automation processes need to be understandable, not a black box, with a clearly agreed margin for error and a system for monitoring and assuring error rates.
- *Trust*: any automated approach needs to be demonstrably trustworthy in arriving at consistently good outcomes.
- *Repeatability*: while the Lexicon-based approach allowed for repetition of the same methodology in any information collection, collections may have unique characteristics that need to be understood and accounted for.

To mitigate the transparency risk, The National Archives’ research team was invited to review this methodology, because it is important to the public, and the records and archives profession, that there is confidence that the right records have been selected, ensuring that government is transparent and accountable. The Cabinet Office algorithm has been published as part of the UK Government’s Algorithmic Transparency Recording Standard, which aims to ensure that algorithmic technologies used by government that replicate human decision-making and that have a direct or indirect effect on the public are capable of scrutiny.^16^

The approach to setting baselines involved a human review of a selection of 1,000 files, selected using systematic random sampling. The disposal decision for each file was recorded with reasons and then the automation was run across the collection and the results compared. In the case of the human assessment, it was decided in retrospect that an incorrect decision had been made in 1% of cases. When we looked at a similar sample of 1000 files assessed by the algorithm, we judged that the algorithm had incorrectly classified files in only 0.4% of cases. Computational approaches to appraisal therefore led to more accurate outcomes. This is consistent with research in other sectors which has shown that, for example, AI reviews of medical scans are at least as good and often more accurate than human reviews (Shen et al. 2019; Nagendran et al. 2020).

To ensure that the Lexicon is relevant, it is updated, or tuned, following a scoping exercise prior to being run across a new collection. Tuning is a process of human review, assisted by search technology to identify likely candidate words or phrases to add to the language model or to adjust weighting.

### Outcomes

Using AI to automate disposal of digital records has provided several benefits. Included in our pilot exercise was a time in motion study of how long it would reasonably take people to read and make a rational decision about the value of a document, then record the outcome. Based on this, it was estimated that a person could potentially review around 200,000 documents a year. At this rate, the Cabinet Office’s legacy collection of over 11 million documents would have taken 59 years to review or they would have had to employ a small army of document reviewers, neither of which was feasible. The AI was able to accurately and rapidly review this volume of documents within their annual disposal cycle, and the cost avoided of not having to employ 59 people over the course of the year was calculated at £2.2 million. The ongoing cost of the entire system, taking account of the technology and the people employed to run it, remains almost £200,000 per annum cheaper than the manual alternative.

### The human element

Discussions about AI often refer to the concept of the humans in the loop, meaning how the rational mind of a person moderates the activity of AI and prevents it from going bad. In designing a new digitally centred process, such as the one described here, the question is also about technology in the loop, or where we can best apply the technology to solve a problem that is beyond human capability to manage. The final decision over whether records are retained or destroyed must remain a human decision because of the legal implications for getting it wrong; we cannot transfer risk to dumb technology. The Cabinet Office’s AI solution is assistive, replicating human analysis and presenting a proposal based on a set of rules that is controlled by human operators.

An extension of the technology-in-the-loop logic is that technology can only properly operate in the way we need it to if it forms a component part of an overall system. The Cabinet Office system has been designed to work regardless of previous systemic failures out of necessity, but ideally the overall records management system in which it operates should include training the human culture to act in particular ways that prevent the system from being undermined by poor or risky behaviour, which may lead to breaches in compliance, inefficiency or real-world damage to the public good. It is notoriously difficult to shape the culture of an organisation around administrative tasks that do not have immediate utility to those charged with carrying them out. Foundational to any records management system is developing a good culture where people value the records they create and carry out some basic, simple and easy to follow actions. This can be summarised as follows:

- Name: all documents will have a name, so a standard for the information elements that should be included in a document name is helpful.
- Store: creating the document in the right place, in an authorised repository, so that it is protected, recoverable and its existence recorded.
- Check: the access permissions required for the document, ensuring that the creator knows who they are sharing it with when they create it in a particular space.
- Share: an extension of “check”, but aimed at ensuring that documents are not created in locked away spaces or silos and information and knowledge is shared widely, and appropriately.

These standards are rigorously enforced, but it has proved vital in making the Cabinet Office’s system, supported by AI, a success. As part of the naming standard, Cabinet Office staff members are required to include the creation date, or another relevant date, such as the date of a meeting, in the international standard; i.e. YYYY–MM–DD. Having this element in the document title provides a backup if the metadata is damaged and provides another data point through which records can easily be identified and sorted using content. This is doubly important when using a language model to power AI. While the AI solution developed by the Cabinet Office helped them to sift records without reliable metadata, it is still important to protect and enhance metadata wherever possible, and the presence of AI in the system provides a feedback requirement for the data (in the form of language) to be consistent, and, ideally, predictable. This requirement may fortify a case for the use of language taxonomies where appropriate, and where it is possible to apply them.

### Where next?

AI is demonstrably capable of taking on onerous or time-consuming tasks that would be practically or economically impossible for humans to undertake alone. The next issue discovered is that the process of tuning the Lexicon is also an onerous task requiring a significant injection of people hours to carry out. This is clearly a target for future AI development: to read a collection and summarise and analyse its content so that language models can be easily updated.

The Cabinet Office solution is focused on reviewing documents in drives, and the mounting numbers of mailboxes archived in their collection has yet to be tackled. Something similar must be possible with email, but it is acknowledged that a different or modified language model to the one currently being used would be required, and that there is more metadata available to inform outcomes; for example:

- the sender and receiver(s) of the message;
- the names of those copied in;
- that the records will range from a single email sent to another person to an extended exchange, often with side chains of correspondence from others (direct recipients or copyees);
- the email header may contain a descriptive title and that this may be helpful and specific in describing the theme of the exchange or obscure or, at worst, misleading;
- the language in the body of the email may be formal or informal, or both at once; e.g. a formal message that contains informalities.

Natural language processing may be appropriate for analysing the text in the body of the email to determine whether the messages are social, ephemeral or corporate, and (if the latter) of value to the historic record or not. In the UK, The National Archives (TNA) has created a LLM Sandbox and carried out research that suggests that decision-makers within an email exchange can be identified by analysing who sends emails to whom (and when), and from this glean some (limited) information about organisational structure. Decision-makers and related emails on a single topic can be identified using an LLM to generate data (“tags”) based on the topics of each email (e.g. “rural school funding”). Using this method, TNA suggests that email networks can be filtered to find (a) the key decision-makers in such areas, and (b) their specific emails relating to those topics.

The approach to filter large bodies of data into smaller classified “chunks” aligns with the review methodology developed by the Cabinet Office and this may provide part of the solution in that it will reduce the volume of material that requires semantic analysis of the main body of text. Multiple approaches can be taken to approach classification problems, but have historically struggled to produce top results on natural language data. The work of TNA appears to show that LLMs can now be used to generate additional, easily vectorised data, to provide key topics and context to any given document. The benefits of this approach are that the classification results are significantly improved.

### Environmental cost

The algorithmic methods of text classification and automated retention decision-making outlined in the article clearly increase document throughput and produce similarly accurate results to a single human reviewer working through a set of records much more slowly. However, AI-assisted review also comes with a significant environmental cost. How can we justify the energy consumption and data storage costs of AI-assisted review? How do these costs compare to the costs of benign neglect, of merely maintaining the “digital heaps” over a longer period? While our own project did not include an assessment of its specific environmental cost, there are benefits from being able to delete ROT information with confidence and thereby reduce data storage and their commensurate energy consumption. As Ian R. Hodgkinson, Nick Jennings and Tom Jackson (2024) point out, AI’s environmental footprint can be significantly decreased “by adopting management strategies prioritising data minimisation, efficient storage, and responsible data disposal.” Recent research has shown that up to 80% of organisations’ on-premise primary business data might be dark data^17^ or ROT data, equating to thousands of terabytes (Jackson et al. 2023). Reducing the prevalence of unnecessary data is therefore essential to limit the environmental cost of the digital revolution.

## Conclusion

For record managers and archivists, AI offers the hope to sort out their “digital heap” and to impose order over chaos. In case of freedom of information requests or public inquiries, the inability to provide requested documents can have severe consequences—from embarrassment to legal liability. Keeping neatly organised records diminishes these risks. As the volumes and complexity of records continue to increase beyond the ability of human records managers and archivists to understand them, computational solutions will increasingly be necessary. Generative and semantic AI solutions will both need to be considered as part of a wider reconsideration of how the practice of records and archives management needs to be redesigned as a digital discipline.

However, as with any technological solution, the application of AI within a business process must be considered carefully, as part of a wider system design. Such designs should consider the extent to which transparency and explainability is important to the user, the wider organisation and any external parties, such as regulators or the public at large. In general, when it comes to the retention and destruction of information, transparency should be default, and algorithms need routine and regular review to ensure they remain fit for the purpose and the information or language model driving them remains correct and relevant.

AI may offer an appropriate solution to fix a problem created through historic issues, such as metadata loss, but it should not necessarily be seen as a panacea to bad practice. The application of AI into a system produces a feedback requirement on all the humans involved in the process to adapt to its presence and to change their behaviours in order to reduce risk and consequently, to improve the performance of the overall system, and prevent future systemic failure. All AI that relies on a language model requires good data to function effectively. A feedback requirement from a semantic AI, such as the one used by Cabinet Office, is an obligation for the organisation to continue to reinforce the creation of good metadata through naming and labelling standards, and potentially by introducing or maintaining taxonomies of language.

While improving current record management practices is necessary, it is important to have realistic expectations, and to remember that perfect order does not exist. In Umberto Eco’s historical novel *The Name of the Rose*, the fictional character William of Baskerville declares: “I behaved stubbornly, pursuing a semblance of order, when I should have known well that there is no order in the universe” (Eco 2014). Although perfect order is an illusion, discoverability is necessary to unlock digital records and enable wide access to users.

To achieve this broad aim of wider discoverability and access, we would like to offer key recommendations to help record managers and archivists apply AI to their collections in a responsible and ethical way:

- *AI needs to be considered as part of an entire system*. An AI system deployed in isolation, without taking into account human behaviours is unlikely to be sustainable. The humans in the system need to be aware of and adapt to the presence of the AI. Indeed, reviewing digital records in the same way as paper records is no longer sustainable due to the volume and complexity of digital recordkeeping systems, and human behaviours need to change and adapt to computational approaches.
- *Senior management buy in is essential*. This could be achieved by explaining the risks of inaction, and by giving estimates of savings made possible by AI.
- *Be bold and join the AI discussion*. Not everyone is an AI expert, but everyone who works with archives and records can join the discussion. Record managers and archivists have advanced expertise on their data, which is essential to tailor AI tools to the specific needs of their sectors.
- *Consult across the archival circle*. In particular, it is important to involve end users of archival collections. Getting regular feedback on their experience is helpful to revise strategies and maximise access to digital collections.

## Data Availability

No datasets were generated or analysed during the current study.

## Footnotes

1 OMB/NARA Memo M-23-07, Update to Transition to Electronic Records, 23 Dec. 2022.

2 “Historically significant” is a fluid concept, and we describe later in the article various criteria generally associated with value in the context of the Cabinet Office. Terms such as “submission,” “minister,” “inquiry,” or the names of international leaders will often point to valuable documents that must be preserved in the long term and made accessible to historians and other users.

3 In a US context, these requests are called Freedom of Information Act (FOIA) requests.

4 The concept of distant reading originates in literary studies. It is generally attributed to Franco Moretti (2000, 2013), and refers to an approach that applies computational methods to literary data for the purposes of literary history and theory. This "distant" approach is often combined with close reading of particularly significant records.

5 The National Archives is part of the government (as a non-ministerial department) and the official archive and publisher for the UK Government, and for England and Wales.

6 Public Records Act 1958, available at https://www.legislation.gov.uk/ukpga/Eliz2/6-7/51/section/3 (accessed 27 Jan. 2025).

7 Code of Practice on the Management of Records issued under Sect. 46 the Freedom of Information Act 2000, 15 July 2021, available at https://www.gov.uk/government/publications/code-of-practice-on-the-management-of-records-issued-under-section-46-the-freedom-of-information-act-2000 (accessed 27 Jan. 2025).

8 “What is GDPR, the EU’s new data protection law?,” available at https://gdpr.eu/what-is-gdpr/ (accessed 27 Jan. 2025).

9 The volumes vary according to format. See the graphic from LDM Global that explains data size in physical equivalence: https://www.ldmglobal.com/wp-content/uploads/161208-aw-am-ldm-global-ediscovery-facts-at-a-glance.pdf (accessed 27 Jan. 2025).

10 “Why Digital Preservation Matters,” available at: https://www.dpconline.org/handbook/digital-preservation/why-digital-preservation-matters (accessed 27 Jan. 2025).

11 *Archives and Manuscripts* 47.3 (2019), available at https://www.tandfonline.com/toc/raam20/47/3; *AI & Society* 37.3 (2022), available at https://link.springer.com/journal/146/volumes-and-issues/37-3; *Archival Science* 22 (2022), available at https://link.springer.com/collections/gdhjcfbdfb; *Journal on Computing and Cultural Heritage* 16.4 (2023), available at https://dl.acm.org/toc/jocch/2023/16/4; *Digital Humanities Quarterly* 18.2 (2024), available at https://www.digitalhumanities.org/dhq/vol/18/2/index.html.

12 https://ai4lam.github.io/awesome-ai4lam/ (accessed 27 Jan. 2025).

13 https://interparestrustai.org/trust (accessed 27 Jan. 2025).

14 Elasticsearch is a free, open-source search and analytics engine that stores, indexes, and analyses data. It is built on the Apache Lucene library.

15 We recognise that this may be a tall order for many organisations regardless of size, staff expertise, training regimes, and resources. However, these recommendations are less time-consuming and more feasible in practice than previous recommendations based on paper records systems.

16 Algorithmic Transparency Recording Standard. Central Digital and Data Office, available at https://www.gov.uk/government/collections/algorithmic-transparency-recording-standard-hub. The specific link for this project at the Cabinet Office can be found here: https://www.gov.uk/algorithmic-transparency-records/cabinet-office-automated-digital-document-review (accessed 27 Jan. 2025).

17 Dark data refers to data that organisations collect, process and store, but only ever use once or never at all.
